# Traumatic irreducible dislocation of the fifth metatarsophalangeal joint in pediatrics: case report and clinical experience

**DOI:** 10.3389/fped.2024.1242082

**Published:** 2024-01-19

**Authors:** Wen Chao Li, Li Liu, Hui Chen, Zhen Dong Wang, Hui Xia Zhou

**Affiliations:** Senior Department of Pediatrics, The Seventh Medical Center of Chinese People’s Liberation Army General Hospital, Beijing, China

**Keywords:** metatarsophalangeal joint, dislocation, osteotomy, surgery, foot

## Abstract

Dislocation of the metatarsophalangeal joint (MTPJ) by trauma commonly occurs in adults. Most dislocations of the MTPJ could be reduced by closed reduction. However, isolated traumatic irreducible dislocation of the fifth MTPJ is an extremely rare injury, particularly in children. We report the case of a 10-year-old boy with irreducible dislocation of the fifth MTPJ who presented with a dorsiflexion injury of the right foot 1 year previously. Closed reduction was attempted but failed. Computed tomography showed the dorsolateral dislocation of the fifth MTPJ. We performed an open reduction and metatarsal bone osteotomy, with a short osteotomy at approximately 0.8 cm. The osteotomy was adjusted to a reduction of the MTPJ and fixation by a lock compression plate. The distal growth plate in the metatarsal bone was protected to avoid pre-closure of the growth plate. There were no instances of dislocation or signs of avascular necrosis of the head of the metatarsal bone. The results of this study demonstrated that open reduction and metatarsal bone osteotomy could be an optional treatment for irreducible dislocation of the fifth MTPJ in children. We should pay more attention to the distal growth plate in the metatarsal bone to avoid pre-closure of the growth plate.

## Introduction

Traumatic dislocation of the fifth metatarsophalangeal joint (MTPJ) is not common, particularly in children (1, 2). When dislocation of the MTPJ is the result of an injury, it usually involves the first MTPJ, while the ﬁfth MTPJ is rarely involved (3). Most MTPJ dislocations could be reduced by close reduction, but this may not be possible in situations that involve delayed treatment with the contracture of the capsule joint or the incarceration of the metatarsal head under the ﬂexor digitorum longus tendon (4). Previous studies showed that dislocation of the MTPJ mainly involved adults; the traumatic irreducible dislocation of the joint in children has rarely been reported (5). Hynes et al. (6) reported that the metatarsal head was incarcerated under the flexor digitorum longus in a case of irreducible dislocation of the fifth MTPJ and returning the flexor digitorum longus could reduce the MTPJ. Boussouga et al. (2) reported a case of dorsal dislocation of the fifth MTPJ in which an open reduction was performed with Kirschner wire fixation to maintain alignment. In the present case, we report a patient with irreducible dislocation of the fifth MTPJ with a failed close reduction, who presented with a dorsiflexion injury of the right foot 1 year previously. It is our goal to introduce a new metatarsal bone osteotomy in children and fixation by lock compression plate.

## Case presentation

**Figure 1:**
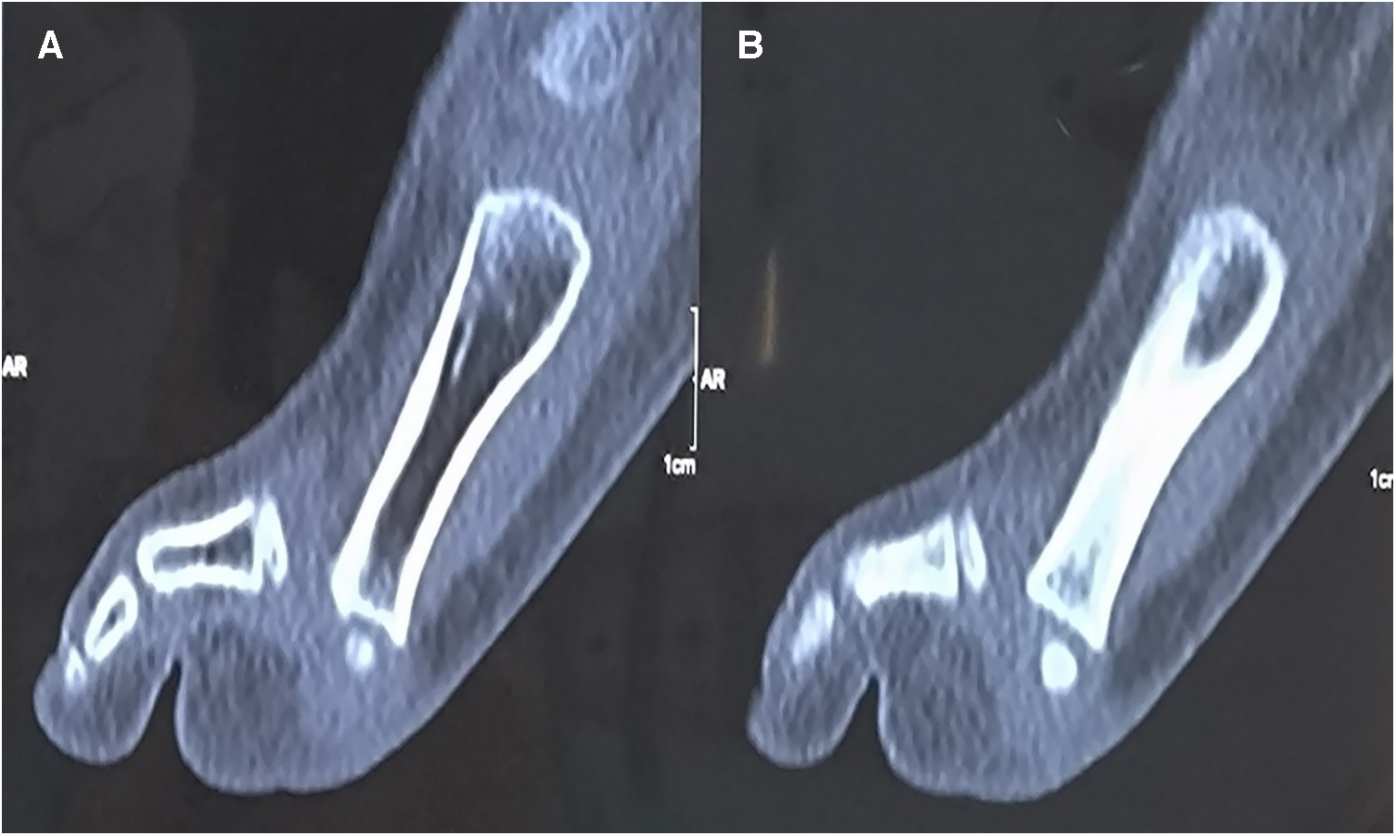
(**A**, **B**) Computed tomography shows the dislocation of the fifth MTPJ in the sagittal image.

**Figure 2:**
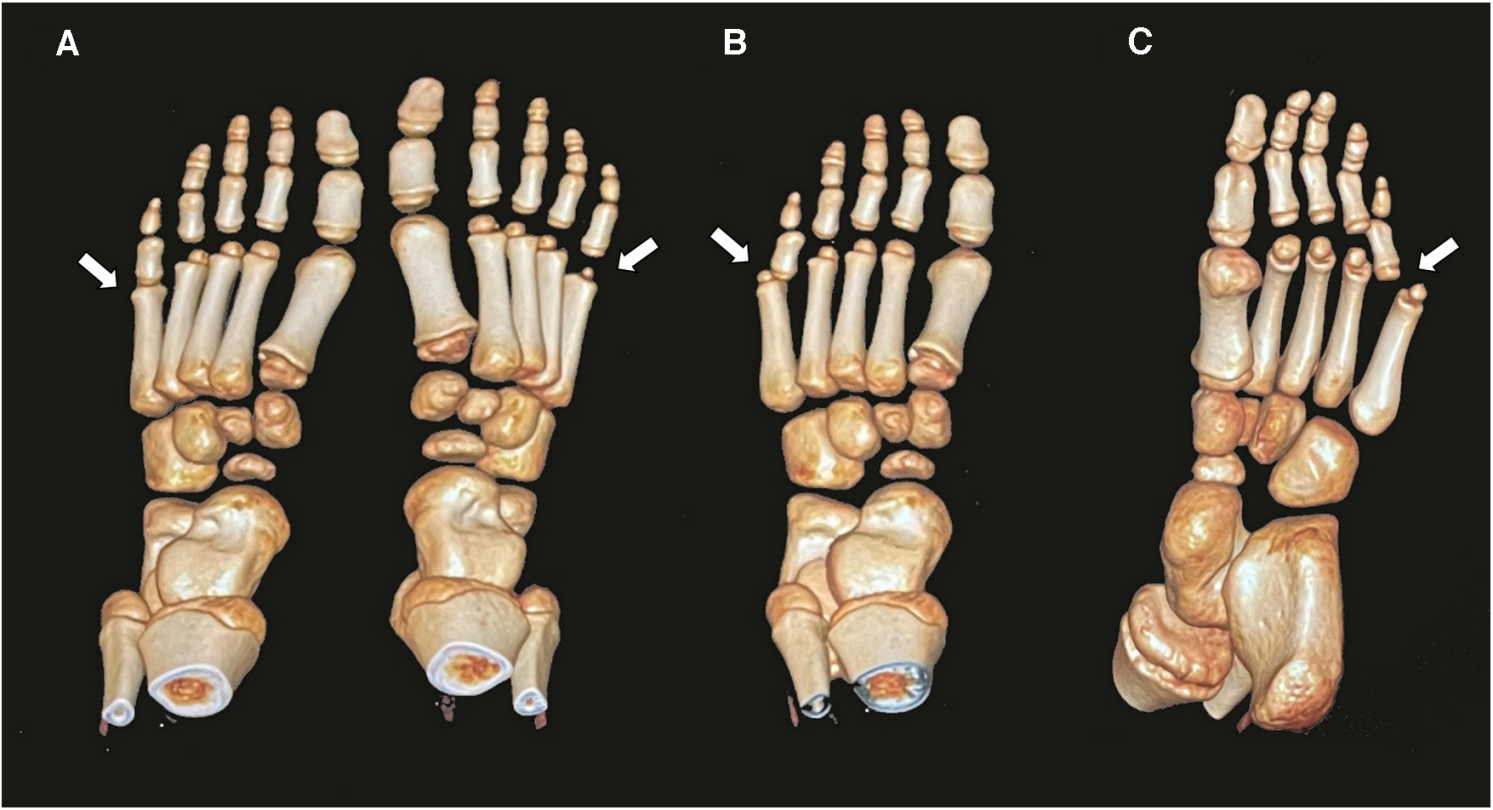
Three-dimensional computed tomography shows the dislocation of the MTPJ and normal MTPJ (**A**); the image in front aspect (**B**); the image in planta pedis (**C**).

A 10-year-old boy presented to the outpatient room with pain in his left foot, limited flexion, and a dislocation of the fifth MTPJ. He had a left toe injury from a fall from a high platform 1 year previously. Unfortunately, he did not receive the correct diagnosis and proper treatment at the time of injury. After 6 months, the boy had experienced limited flexion of the fifth MTPJ and dislocation of the joint, determined by a physiological examination. At that time, the attempted closed reduction of the joint was performed with a failed result. On clinical examination, the patient had deep tenderness on the plantar aspect of the fifth MTPJ with limited movements. The MTPJ had capsule laxity but could not be reduced manually. Computed tomography (CT) showed a dorsolateral dislocation of the fifth MTPJ (Figures 1, 2).

Using general anesthesia and ﬂuoroscopic imaging, a further attempt at closed reduction failed. Faced with the traumatic irreducible dislocation of the joint, we decided to perform an open reduction and short osteotomy of the metatarsal bones. The child was placed in a supine position with a thigh tourniquet (200 mmHg).

**Figure 3:**
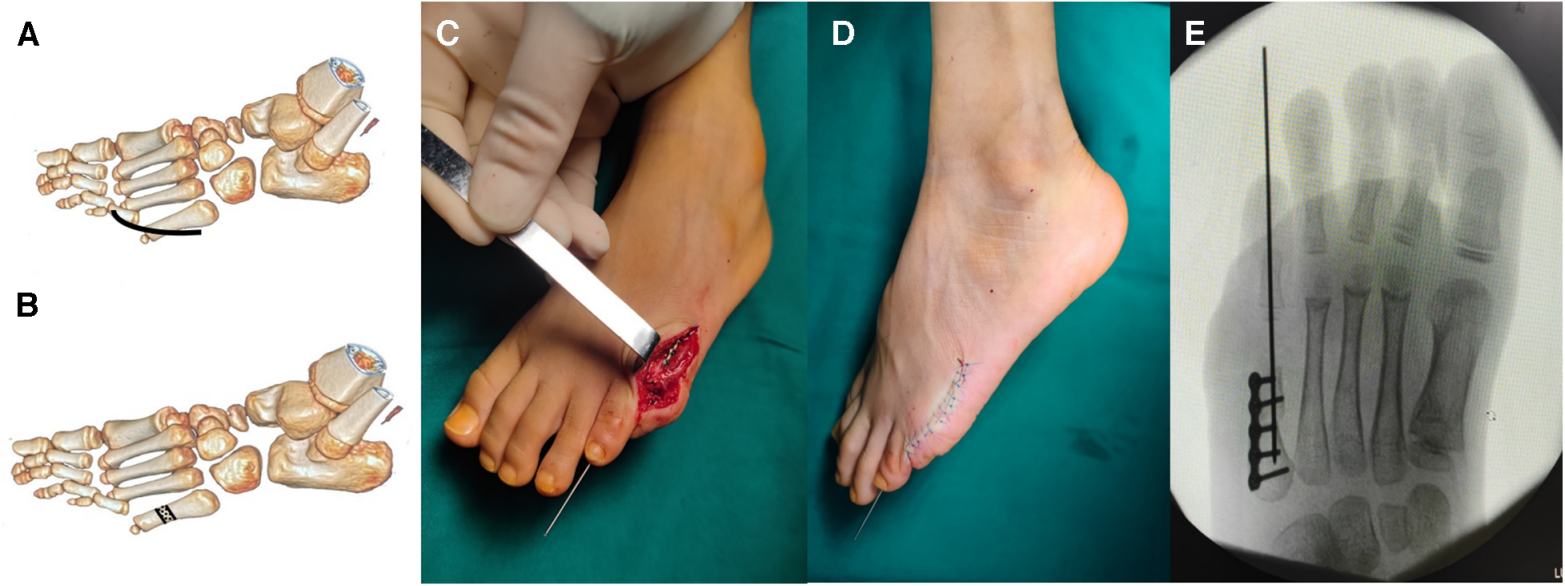
The metatarsal osteotomy procedure. The curvilinear incision was performed over the fifth MTPJ (**A**); the osteotomy in the metatarsal bone (**B**); after reduction of the MTPJ, the osteotomy was performed with fixation by the lock compression plate (**C**); the Kirschner wire was used to keep the stability of the MTPJ (**D**); the anteroposterior radiograph shows the reduction of the MTPJ (**E**).

We performed a single, curvilinear incision approximately 3 cm over the MTPJ to reduce dorsal contracture. Then, the subcutaneous tissue was separated to expose the fifth MTPJ. The capsule of the joint was cut open to expose the head of the fifth MTPJ and the synovial tissue was removed. After the failed closed reduction of the MTPJ, the metatarsal bone osteotomy was done using a micro-oscillating saw to avoid injury to the growth plate, and the bone was shortened by approximately 0.8 cm. The osteotomy position was adjusted to reduce the MTPJ and fixation was achieved by a lock compression plate. Then, the capsule of the joint was sutured with absorbable sutures, and a Kirschner wire was used to maintain the stability of the MTPJ (Figure 3). The incision was then irrigated with sterile saline and closed.

Postoperatively, the non-weight brace was used, and analgesia and anti-infection were provided. Full weight-bearing was advised at 6 weeks. The osteotomy led to bone union after the 10-week follow-up. The patient has a normal movement of the MTPJ without pain. The X-ray at the follow-up showed a reduction of the fifth MTPJ.

## Discussion

Dislocation of the MTPJ mainly involves the forced extension of the foot, which is consistent with the injury sustained by the patient (7, 8). The dislocation of the MTPJ is mainly reported as a result of road trafﬁc accidents, sporting accidents, falls, and the dropping of heavy objects on the foot. The lesion results in either hyperextension or hyperﬂexion of the MTPJ. The direction of the dislocation is most frequently dorsal as a result of the forced hyperextension of the joint (2). The ﬁbrocartilaginous plantar plate located at the metatarsal head could endure compressive and tensile forces and increase joint stability. It is well known that the MTPJ is protected by an articular capsule and its associated collateral, dorsal, and plantar ligaments (1). Dislocation of the MTPJ may be a result of trauma, with injury to the plantar plate and collateral ligaments. Then, the plantar plate located around the metatarsal head could endure the compressive and tensile forces with the added joint stability (9, 10). In cases of dislocation, the excessive dorsiﬂexion force on the foot causes the metatarsal head to be pushed in a plantar direction during walking or running, which may cause foot pain (11). The force could destroy the plantar plate and aggravate the dislocation of the MTPJ.

Previous studies reported that most dislocations of the fifth MTPJ could be closed using closed reduction; however, cases involving incarceration of the head of the metatarsal under the flexor digitorum longus tendon may be performed using open reduction (2, 6). We applied a dorsal approach over the fifth MTPJ, which could be more appropriate in the case involving dorsal dislocation of the MTPJ in toe deformity. Some authors suggested that the dorsal approach should be performed in the surgery (1). During the operation, we found that the metatarsal head was dislocated in the plantar direction, exposing the articular surface of the proximal phalanx. Then, the interposed plantar capsule obstructing the reduction of the MTPJ should be excised. However, some studies showed that the plantar plate, formed by the plantar aponeurosis and the plantar joint capsule, plays a major role in the dorsoplantar stability of the MTPJ (12). These authors have usually performed the plantar approach and have suggested that the approach could permit a re-approximation of the deep transverse intermetatarsal ligament and plantar plate (13).

In the surgical treatment of dislocations of the MTPJ, there have been dorsal soft tissue release with K-wire fixation, metatarsal neck osteotomy, and silicone implants. Metatarsal bone osteotomy plays an important role in the open reduction of MTPJ dislocation. Helal (14) described a simple oblique osteotomy of the metatarsal neck in the treatment of metatarsalgia that did not require internal fixation. Helal and Greiss reported that shortening the metatarsal without touching the joint capsules could promote the correction of the MTPJ dislocation (15). Trnka et al. (16) also reported the Helal osteotomy and Weil osteotomy in the treatment of correction of subluxation or dislocated MTPJ.

The Weil osteotomy was usually used in irreducible dislocation of the MTPJ and metatarsalgia and was recommended as a safe and reproducible approach for correction of the lesser ray (12, 17). This osteotomy is an oblique osteotomy of the metatarsal head and neck, parallel to the ground surface, which controls the shortening of the metatarsal bones by internal fixation with screws or pins (18). However, the metatarsal neck osteotomy in a child could injure the proximal growth plate of the metatarsal bone. In our study, we performed the metatarsal bone osteotomy far from the growth plate and shortened the bone to reduce the MTPJ. With the contracture of ligaments and the joint capsule, the metatarsal bone was shortened by 0.8 cm in order to maintain the reduction of the MTPJ. At the follow-up, the shortened metatarsal bone did not influence the biomechanics of the foot. The osteotomy of the bone was adjusted to maintain the reduction of the MTPJ and fixation was achieved by a lock compression plate instead of a screw in the Weil osteotomy. In our case, the osteotomy resulted in a shortened metatarsal bone without limiting the MTPJ.

Traumatic dislocation of the fifth MTPJ is a rare injury, particularly in children. The traditional Weil osteotomy is mainly applied in dislocations of the MTPJ in adults. The distal growth plate in the metatarsal bone prevents the application of the Weil osteotomy in children. We performed an open reduction and metatarsal bone osteotomy in the irreducible dislocation of the fifth MTPJ in a child. We should pay more attention to the distal radius growth plate in the metatarsal bone to avoid pre-closure of the growth plate. To the best of our knowledge, this is the first report of irreducible dislocation of the fifth MTPJ and open reduction with plate fixation in a child.

## Data Availability

The original contributions presented in the study are included in the article/Supplementary Material, further inquiries can be directed to the corresponding authors.
